# Use of Black Poplar Leaves for the Biomonitoring of Air Pollution in an Urban Agglomeration

**DOI:** 10.3390/plants10030548

**Published:** 2021-03-14

**Authors:** Levente Levei, Oana Cadar, Vanda Babalau-Fuss, Eniko Kovacs, Anamaria Iulia Torok, Erika Andrea Levei, Alexandru Ozunu

**Affiliations:** 1 INCDO-INOE 2000, Research Institute for Analytical Instrumentation, 67 Donath Street, RO-400296 Cluj-Napoca, Romania; levente.levei@icia.ro (L.L.); vanda.fuss@icia.ro (V.B.-F.); eniko.kovacs@icia.ro (E.K.); iulia.torok@icia.ro (A.I.T.); erika.levei@icia.ro (E.A.L.); 2 Faculty of Environmental Sciences and Engineering, Babes-Bolyai University, 30 Fantanele Street, RO-400294 Cluj-Napoca, Romania; alexandru.ozunu@ubbcluj.ro; 3 Faculty of Food Science and Technology, University of Agricultural Science and Veterinary Medicine, 3-5 Calea Manastur Street, RO-400372 Cluj-Napoca, Romania; 4 Faculty of Horticulture, University of Agricultural Science and Veterinary Medicine, 3-5 Calea Manastur Street, RO-400372 Cluj-Napoca, Romania; 5 DIMTEC, University of the Free State, Nelson Mandela Street, Bloemfontein SA-9300, South Africa

**Keywords:** metals, PM_10_, PM_2.5_, PM_1_, *Populus nigra*

## Abstract

Trees are considered to be an effective tool for metal pollution biomonitoring. In the present study, the concentration of metals (Cu, Pb, Zn, Cd, Co, Ni, Fe, Mn, and Al) in black poplar leaves (*Populus nigra* L.), together with the concentration of PM_10_, PM_2.5_, PM_1_, and total suspended particles (TSP), was used for the air pollution biomonitoring in 12 sites from various areas of Cluj-Napoca city, Romania. The concentration of PM_10_ in the air was high, but their metal content was low. However, Cu, Pb, and Zn were moderately enriched, while Cd was highly enriched in PM_10_ due to anthropogenic sources. The average metal concentration in leaves decreased in the order Zn>>Fe>Mn>Al>Pb>Ni>Cu>Co>Cd and increased with the increase of PM_10_ concentration, indicating that poplar leaves are sensitive to air pollution. The principal component analysis indicated that traffic, waste burning, road dust resuspension, and soil contamination are the main anthropogenic sources of metals in poplar leaves. The results indicated that black poplar leaves are a suitable biomonitoring tool for metal pollution, in urban environments.

## 1. Introduction

Metals are important constituents for a wide range of commodities in our daily life, therefore, every human activity that produces and uses these commodities has the potential to release metals into the environment [1]. Pollution with metals negatively impacts both the ecosystems and human health due to its high toxicity, high bioaccumulation rates, and low biodegradation potential [2,3,4]. 

Air pollution is recognized as one of the factors that decrease life expectancy and increase the incidence of cardiovascular and respiratory pathologies as the fine and ultrafine particulates can penetrate deeply into the lungs [5,6]. Air pollution with particulate matter (PM), with aerodynamic diameters <10 µm (PM_10_), 2.5 μm (PM_2.5_), and 1 μm (PM_1_) are the main challenges, especially in urban agglomerations, where besides the industrial activities, vehicular traffic (exhaust emissions, tire and brake wear, mechanical frictions, and lubricants), surface runoff, re-suspended dust, and fossil fuel combustion are considered important pollution sources [7,8,9]. Depending on its diameter, PM released into the atmosphere can travel high distances, has a high airborne lifetime, and may suffer various transformations [10]. Once deposited on surfaces, it can be relocated and dispersed by wind and runoff [7,11]. PM_10_ can contain toxic metals and organic compounds [12,13].

Vegetation, especially trees, are considered ecosystem service providers as they can reduce the pollutants level in urban areas by removing gaseous air pollution or intercepting airborne particulates [4,14]. A decision-making structure for the selection of the most suitable types of trees for urban areas was developed by Vlachokostas et al. [15]. Exogenous factors, such as the PM diameter and shape, exposure duration and spatial distribution, environmental variables (wind and temperature) might influence the PM deposition and metal accumulation capacity, but intrinsic features of the plant such as foliage type (deciduous, evergreen), morphology and texture of the leaves and respiration’s rate determine the trees sensitivity to pollutants [11,12]. The leaves are the main sinks for metals, being more sensitive to pollution than the other plant parts (flowers, fruits, bark).

Metal pollution biomonitoring using trees is based on the changes determined by toxic metals on the morphology or composition of the tree and provides information on the contaminant’s concentration and spatial distribution in the urban environment, at a relatively low cost [8]. Trees can reflect the metal pollution both in soil and atmosphere [1,7,14,16,17,18,19], being able to biomonitor metal pollution over long periods, to quantify pollution parameters and to identify the spatial and temporal distribution patterns of the pollutants [7,14]. The metals reach plants through soil, water, and the atmosphere. In the case of trees, the metal uptake takes place in the leaves and roots systems and is controlled by the species characteristics, the concentration of metal, as well as the solubility and bioavailability of the metals. Generally, it is difficult to discriminate between the uptake routes of metals and more difficult to determine whether the uptaken metal originates from the air, water, or soil. However, in the case of trees, the metal uptake by leaves is considered the main uptake route. Tomasevic et al. reported that Cu, Pb, Zn, and Cd in linden and horse chestnut leaves from city parks accumulate mostly following atmospheric deposition [20]. The suitability of poplar leaves to reflect the atmospheric Hg contamination was reported by Assad et al. [21]. Molnar et al. used common hackberry (*Celtis occidentalis*) and common lime (*Tilia x europaea*) leaves to assess the level of air pollution (in urban, industrial, and rural areas of Debrecen, Hungary [9].

The poplar (*Populus* spp.) is one of the most effective biomonitors among the tree species due to its leaves’ high sensitivity to metal pollution [22]. Poplar is a perennial deciduous tree species belonging to the Salicaceae family. It is characterized by fast growth, extensive root system, large biomass production, high resistance to water and nutrients scarcity, storms and cold weather, and also by its high metal tolerance and accumulation potential [23,24]. It accumulates metals through the root system and the foliage, then, by translocation, high metal contents are stored in the leaves [25,26]. As poplar species can easily reach 25 m heights and have the right stems, they are frequently used in landscaping or as green screens.

The response to environmental stressing conditions varies from plant species to plant species. High metal contents also act as stressors and trigger different protection strategies in plants. These strategies involve a complex interplay of physiological and biochemical processes, gene expression, changes in metabolism, and protein synthesis. In the case of the poplar, regulation of the metal’s homeostasis by reduced uptake or increased excretion, detoxification and translocation are the main protection mechanisms [27,28].

There are more than 50 poplar varieties, but the most spread species in Europe are the black poplar (*Populus nigra* L.), white poplar (*Populus alba* L.), aspen (*Populus tremula* L.), and several species of poplar clones. The black poplar is one of the tree species used for air quality biomonitoring near roads or in urban areas as it is widely spread, in both urban and industrial environments, providing a large leaf area for pollutants absorption and accumulation [17]. It is more sensitive to lead stress than other poplar species [29]. Numerous studies reported the poplar’s efficiency in phytoremediation and the effect of metals on the poplar’s physiological processes. Regarding the poplar’s biomonitoring capabilities, most of the research focuses on their use in soil pollution and only a few in air pollution [16,17,22,23,30,31,32,33,34,35,36,37].

The objective of the study was to determine the metal content of poplar leaves in Cluj-Napoca city, Romania, and to assess the potential of poplar leaves to be used for biomonitoring air pollution with particulates in urban environments. The concentration of metals in the poplar leaves from 12 sites situated in various regions of Cluj-Napoca city was correlated with the concentration of PM_10_, PM_2.5_, PM_1_, and total suspended particles (TSP) measured in each site. The metal content of PM_10_ was measured in one of the sites. Multivariate statistics were applied to identify the sources of metals in Cluj-Napoca city.

## 2. Results and Discussion

### 2.1. Particulates in Air

**Figure 1:**
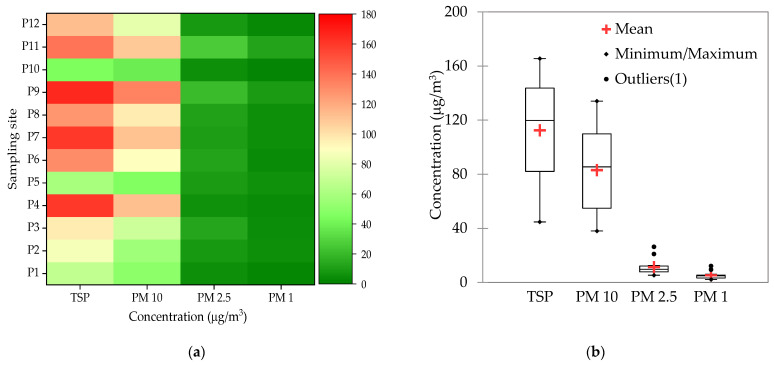
Total suspended particles (TSP), PM_10_, PM_2.5_, and PM_1_ in ambient air in the study sites: (**a**) Concentration; (**b**) Minimum, mean, and maximum concentration.

The TSP and PM_10_ concentrations in the 12 studied sites from Cluj-Napoca city varied widely from site to site, during the same day, while the PM_2.5_ and PM_1_ were, with few exceptions, comparable in all the sites (Figure 1). 

The highest PM_10_ and TSP were measured in site 9, while the highest PM_2.5_ and PM_1_ were found in site 11, both sites being characterized by intense traffic and by the presence of construction sites. The lowest values were measured in site 10, considered a reference site, being situated in a green area with relatively low traffic. The TSP values were above the maximum admitted level (150 μg/m^3^) in 3 sites, the PM_10_ concentrations exceeded the daily limit value (50 μg/m^3^) in 9 of the studied sites, while the PM_2.5_ was below the annual limit value (25 μg/m^3^) in all sites, except one. While the average TSP (113 μg/m^3^) was below its maximum admitted threshold, the average PM_10_ (83 μg/m^3^) exceeded its limit.

**Figure 2:**
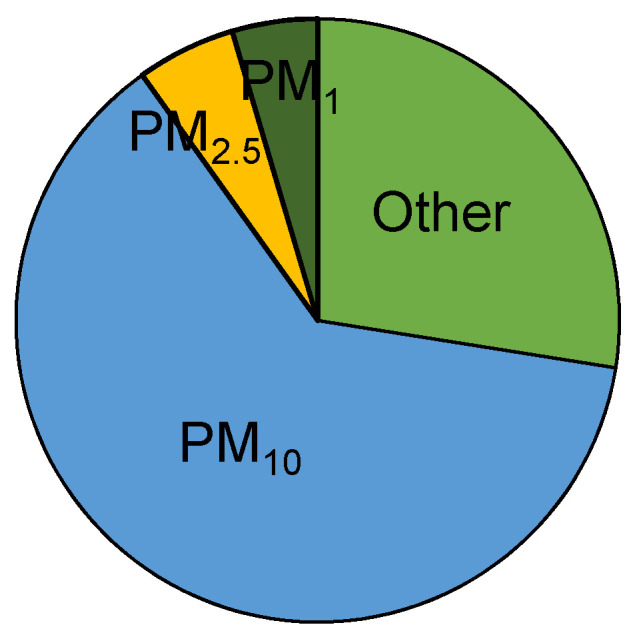
Average % distribution of suspended particles fraction.

The daily PM_10_ measured during 7 consecutive days starting from the leaves sampling day with the high-volume sampler on filters also showed high PM_10_ values on the day of leaves sampling (day 1). However, the values sharply decreased from day 1 (90 μg/m^3^) to day 7 (13 μg/m^3^), indicating a high variability of PM_10_ between consecutive days in the same site (site 11). The daily limit value (50 μg/m^3^) set by the Air Quality Directive (2008/50/EC) for PM_10_ was only exceeded on day 1, the measured value decreasing to 44 μg/m^3^ the next day [38]. The average TSP distribution among fractions (Figure 2) showed that PM_10_ was the major fraction (63%), while PM_2.5_ and PM_1_ represented 5.3% and 4.6%, respectively.

### 2.2. Metal Concentration in PM_10_

**Figure 3:**
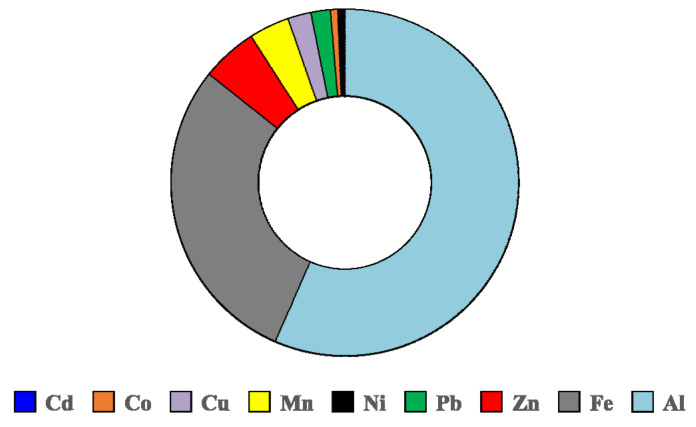
Percentage distribution of metals in the PM_10_ fraction.

The content of metals in PM_10_ in site 11 was low and varied slightly during the 7 monitored days. The average concentrations of metals associated to PM_10_ were: Cd 0.05, Co 0.43, Cu 1.05, Mn 1.88, Ni 0.24, Pb 0.76, Zn 2.55, Fe 95, Al 37 ng/m^3^. None of the legislated reference thresholds set by the Directive 2008/50/EC (Pb 0.5 μg/m^3^) and by the Directive 2004/107/EC (Ni 20 ng/m^3^ and Cd 5 ng/m^3^) were exceeded [38,39]. The high concentrations of Fe and Al compared to the other metals indicated different sources, most probably they originated from the soil particles mobilized from roadsides by the traffic [12]. The other metals could have originated mainly from vehicles’ exhaust, road dust, break and tire wear, but also from fossil fuel combustion [40]. The 9 determined metals represented less than 0.4% of the PM_10_, the highest percentage corresponding to Al (0.19%) and Fe (0.10%), the rest of the metals accounted for only 0.07% (Figure 3).

The concentrations of Cd, Ni, Zn, and Pb were lower, while Al concentration was higher in Cluj-Napoca than in Ahvaz city, Iran [41]. The contents of Zn and Ni were slightly higher in Cluj-Napoca (Romania), while that of Pb, Cu, and Cd associated with PM_10_ were comparable with those reported for Varanasi (India) [42]. Our values were much lower than those obtained in the industrial city of Acerra (Italy) [43].

The results showed that the enrichment factor (EF) of Co, Mn, Ni, and Fe in PM_10_ did not exceed 10, being most probably related to the earth’s crust and not to anthropogenic sources. The enrichment of PM_10_ in Pb, Cu, Co, Zn, Cd, V, Ni was also noticed in the cities of Istanbul, Turkey, and Acerra, Italy [43,44,45]. Cu (EF_PM10_ = 16), Pb (EF_PM10_ = 18), Zn (EF_PM10_ = 16) were found to be moderately enriched and of mixed origins, while Cd (EF_PM10_ = 230) was highly enriched, and thus of anthropogenic origin. These results are in line with those reported by Tomasevic et al., who found that atmospheric deposition in urban parks from Belgrade was enriched with Cu, Pb, Zn, and Cd [20].

### 2.3. Metal Concentration in Poplar Leaves

**Figure 4:**
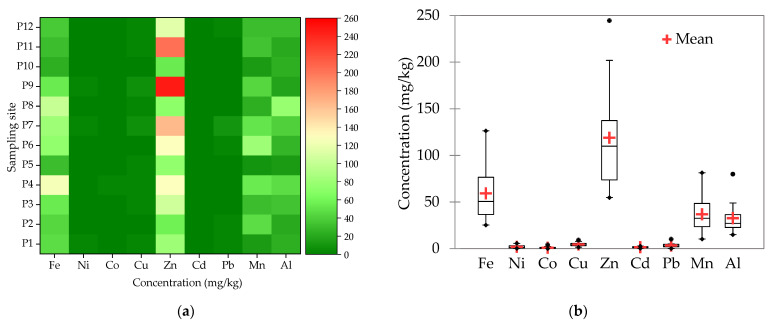
Concentration of metals in poplar leaves: (**a**) Concentration; (**b**) Minimum, average, and maximum.

The metal concentrations in poplar leaves widely fluctuated (Figure 4), however, the phytotoxic level was not reached for any of the analyzed metals. Fe, Mn, Cu, Zn are considered micronutrients with important roles in plant’s growth but may become toxic at high concentrations, while the role of Al, Pb, Ni, Cd, and Co do not have a known role in plants’ physiology and are toxic [33]. The highest variability was found for Zn, which was also the major element in poplar leaves. Generally, Zn in plants ranges between 10 and 160 mg/kg and becomes toxic in the range of 70–400 mg/kg [46,47]. In 3 of the studied sites, the Zn concentration of poplar leaves was found to be higher than the normal range.

This fact could be a consequence of the high Zn content (2.7 ng/m^3^) found to be associated with PM_10_ or of the poplar’s ability to preferentially accumulate Zn [48]. Moreover, at low contents, Zn has an important role in the plant’s growth, being an important constituent of several proteins and enzymes [49]. Another micronutrient for plants is Cu, which is normally found in the range of 0.4–45.8 mg/kg in plants, becoming toxic at higher levels [47]. At all the studied sites, Cu in leaves had low variability, its concentration being found in the normal range, in agreement with the low Cu contents found to be associated with PM_10_. The lowest variability was found for Cd, where the measured values ranged between 0.3–2.7 mg/kg, in agreement with the low concentration of Cd (0.05 ng/m^3^) found to be associated with PM_10_ and with the usual Cd concentration found in plants (<10 mg/kg) [20]. Similar to Cu and Cd, Pb had low concentrations in the poplar leaves, being <3 mg/kg in the majority of the samples, far below the toxic levels (>20 mg/kg) [47]. A possible cause for the low Pb content in leaves could originate from the fact that the Pb uptake occurs through the root system and not by particulates deposition. Moreover, Pb translocation to the aerial parts is usually low [33].

Fe and Al were found in lower concentrations than Zn, but in higher concentrations than the other studied metals. These elements generally come from soil particles dispersed by winds and traffic or from fossil fuel burning, followed by a subsequent deposition on the leaves [40,50]. The concentrations of Fe, Al, Mn, and Co were also lower than the typical range found in plants: 640–2486, 200–1000, 15–100, and 0.1–10 mg/kg, respectively [47]. Of these metals, Fe and Mn are considered essential microelements for plant growth, while Co has no proven physiological function [47]. According to Hajar et al., the typical concentration range for plants is 0.1–3.7 mg/kg [47]. The average Ni content in *P. nigra* leaves (2 mg/kg) obtained in the present study was within the typical concentration range, except for 3 of the sites, where it was slightly higher. The concentration of metals in poplar leaves collected from the reference site (site 10) was the lowest for Fe, Ni, and Zn, but in the case of the other elements, lower values were found in some of the other sites. The concentrations of Cu, Ni, Pb were comparable, while those of Zn and Cd were slightly higher than those found in poplar leaves collected in Navodari, Romania [32]. Similar contents of Cd, Ni, Cu, and Pb and much higher values of Mn, Fe, and Zn were reported in white poplar leaves collected in a spill that affected the mining area of the Guadiamar basin, Spain [33]. The concentrations obtained in our study were higher for Zn, Cu, and Cd, and comparable for Pb with those determined in the leaves of *Populus deltoides* in Yazd city, Iran [46]. The average Zn content was 4 times lower than the concentration determined in black poplar leaves from the heavily industrialized Ust-Kamenogorsk city in Kazakhstan [22]. In comparison with *P. nigra*, the leaves of local pine trees, *Pinus eldarica*, from Yazd city, Iran, accumulated Fe in high concentrations (345 mg/kg) and Cd in low concentrations (0.73 mg/kg). Cu concentrations were in the normal range (30 mg/kg), except for one sampling site [51]. In a study carried out by Norouzi et al. [52], *Platanus orientalis* leaves were used as a bioindicator of atmospheric pollution, by determining the concentrations of Cu, Fe, Mn, Ni, Pb, and Zn. Their results indicated significant levels of Fe and Mn, followed by Zn, Cu, and Ni, and a low level of Pb (Table 1).

**Figure 5:**
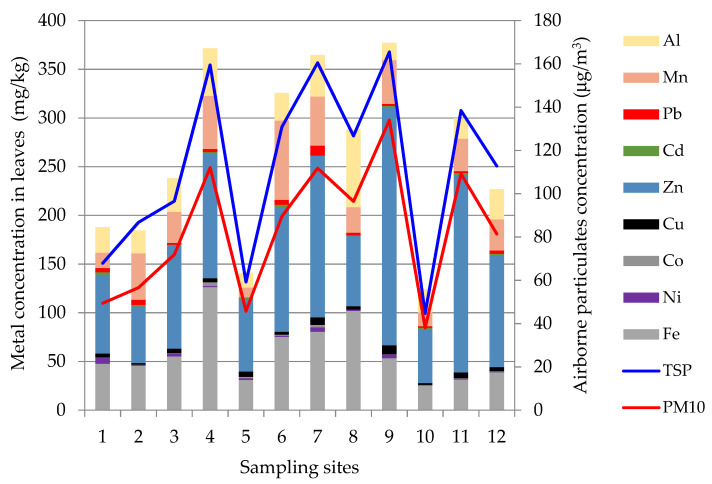
Concentration of metals in poplar leaves (mg/kg) and of TSP and PM_10_ (μg/m^3^) in air.

The total metal content in poplar leaves raised with the increase of PM_10_ and TSP (Figure 5), although a linear relationship was not observed. The lowest metal contents were found in site 10, considered as a reference site for Cluj-Napoca. The highest total metal content in leaves was found in sites 4, 7, and 9, together with the highest levels of TSP and PM_10_. These sites are characterized by intense traffic and proximity to the tram or train lines. The average total metals content in poplar leaves was 46% for Zn, 23% for Fe, 14% for Mn, and 12% for Al. Although metals emitted by urban pollution sources can easily bind to airborne particulates and then can be deposited on the leaves’ surfaces, their accumulation in the poplar leaves also depends on the meteorological conditions (wind, rain) and the individual tree physiology. The high concentrations of Zn found in the leaves suggest that poplar is a good accumulator and can be used not only for biomonitoring but also as a natural barrier, useful for contaminants retention in vulnerable areas. Our findings are in agreement with those of Liu et al., who reported the suitability of *Populus tomentosa* for the reduction of Zn from airborne dust [48]. Similar to other tree species, Zn is an essential element for poplar, but in excess, it may block the absorption of other essential metals (P, Mg, Mn) and trigger toxic effects such as growth inhibition and leaf chlorosis. These effects were more visible in the early development stages of poplar than in mature plants, where the regulator mechanisms were fully developed. The homeostasis of Zn was assured by the active metabolism in roots and leaves. The Zn transport and accumulation were regulated by an 1186-amino acid P-type heavy metal ATPase (HMA4) while Zn detoxification was regulated by phytochelatins and metallothionines [27].

The metal accumulation index (MAI) for poplar species from Cluj-Napoca was 1.7, showing that poplar is a moderate metal accumulator. The obtained MAI value could be explained, on the one hand, by the low metal content in the air and, on the other hand by the tolerance mechanisms developed by poplar [26]. The MAI for black poplar in this study was lower than for *Populous deltoids* (MAI = 6.6) and *Populus tomentosa* (MAI = 7.1) [46,55]. 

The metal accumulation factor (MAF) calculated as the ratio between the metal content in the poplar leaves from the study site and from a reference site (site 10 in our study) showed that metal accumulation occurred in each site, for all the studied metals, except for Cd. Average MAF > 2 were obtained for Zn (2.3), Pb (2.4), Fe (2.5), Cu (2.5), Mn (2.8), Co (3.8), and Ni (6.8). The lack of Cd accumulation in all sites could be explained by the high content of Cd found in the poplar leaves from the reference site and by the low intrinsic ability of poplar to bioaccumulate Cd compared to other metals. This fact could suggest a wider spread of Cd pollution than expected, that also reached green areas situated far from the intense traffic or a possible transfer from the soil. 

Our data indicated no enrichment for Cd, minor enrichments for Fe, Pb, Cu, Co, Zn, Mn, and a moderate enrichment for Ni. The spatial distribution of both MAF and EF_poplar_ varied from metal to metal suggesting the existence of local, unidentified pollution sources that may overlap with the pollution sources related to traffic.

### 2.4. Metal Sources 

The principal component analysis (PCA) revealed that 4 principal components (PC’s) with eigenvalues higher than 1 explain 87% of the system variability (Table 2). The PC1 explains 38.3% of the variability and is related to the positive loadings of Cu, Zn, and particulates and could be associated with air pollution, most probably from traffic and combustion processes, as Zn is an indicator of tire and brake wear and waste burning [56]. PC2 explains 25.9% of the variability and includes Fe, Co, Pb, Mn, TSP and could be associated with air pollution with coarse particles due to road dust resuspension. The influence of these two PC’s is confirmed by the high enrichment of Cu, Pb, and Zn in PM_10_. PC3 explains 12.3% of the variability and includes Ni, Cu, and Pb and could be attributed to soil pollution. The PC4 explains 10.2% of the variability and groups the Fe and Al with positive loading and could represent natural sources, such as earth crust erosion.

**Figure 6:**
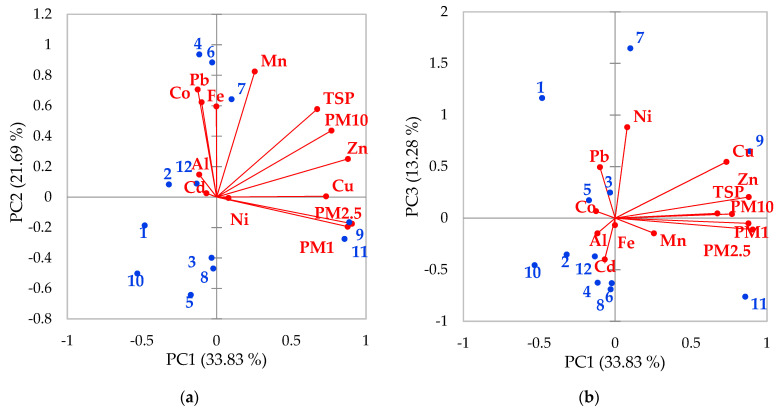
PCA biplots representing both observations (sampling sites) and variables (metal content in poplar leaves and PM concentration in air): (**a**) PC1–PC2, (**b**) PC1–PC3.

Wilks [57] explains the PCA technique as a tool for lowering the dimensionality of a model (data compression) by grouping the initial variables into eigenvectors (linearly independent dimensions). The biplot is a graphical method for representing these dimensions two by two, allowing to visualize the influence (projections) of all the initial variables on the eigenvectors. A biplot was used to simultaneously represent the observations from the 12 sampling sites and the variables: the Fe, Ni, Co, Cu, Zn, Cd, Pb, Mn, Al content in poplar leaves and PM_1_, PM_2.5_, and PM_10_ concentrations and TSP in the air. The sampling sites are grouped according to Figure 6 into 3 groups: G1 contains sites 9 and 11 situated in an area with a high concentration of particulates where the poplar leaves had high Cu and Zn and low Mn, Pb, Fe, Co concentrations, situated under the influence of intense vehicular traffic and construction sites. 

G2 groups at sites 4, 6, 7, that had low PM_1_ and PM_2.5_, but high TSP PM_10_ and Fe, Co, Pb, Mn in poplar leaves were impacted by dust resuspension. G3, which contains the other sites, was characterized by low levels of air particulates and low levels of metals in the poplar leaves. These sites are situated at higher distances from the roads with intense traffic. Our data indicate that poplar is a good passive biomonitor of the spatial distribution of metal pollution and may be used in further urban greening programs for biomonitoring, as well as for air pollution reduction purposes.

## 3. Materials and Methods

### 3.1. Sampling and On-Site Measurements

Situated in the north-western part of Romania, Cluj-Napoca city is one of the most populated cities in the country, with around 420,000 inhabitants in the metropolitan area. The city is located in the Somes Mic river valley (average altitude 363 m) and is bordered in the west by the Hoia hill (507 m) and in the south by the Feleacu hill (759 m). Cluj-Napoca has a continental climate with warm, dry summers and cold winters. The average annual temperature is 8.2 °C and the average precipitation is 557 mm. The dominant wind directions are NW (~15%), NE (~12%), W (~10%), and SW (~10%). The temperatures are slightly lower in the parts of the city situated along the Somes Mic river than in the rest of the city, as thermal inversions are more frequent along the river. In the city, there are registered around 1200 buses, 150,000 passenger cars, 25,000 freight vehicles, and 8000 mopeds and motorcycles. The city has low industrial activity, the main industrial polluting sources being progressively replaced by services and IT companies, in the last decades. The city is short of green areas and parks, the land use nearby the city being changed from agricultural land into residential locations by the intense urban development. The main metal pollution sources are mostly related to traffic, construction sites, urban runoff, residential heating, and municipal landfilling [58].

Poplar is a frequent tree species in Romania, planted mostly at the beginning of the 19th century, mainly along roads and railroads for landscaping purposes. Currently, most of these trees were cut, partly due to their aging and partly as a consequence of the city’s expansion, road enlargement, and rehabilitation. Nowadays, in Cluj-Napoca, poplar is mainly found in small green areas along the Somes Mic River bank, small roads, or buildings.

**Figure 7:**
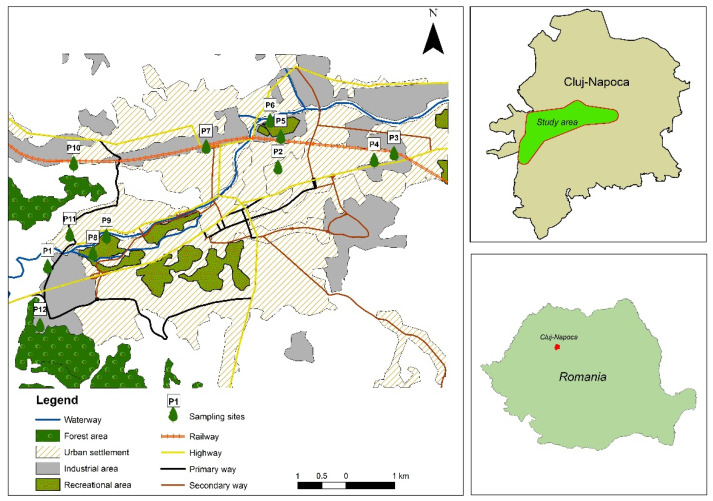
Map of study area and sampling points.

In September 2018, after 10 days without precipitations, black poplar (*Populus nigra* L.) leaves were collected from 12 sites situated in Cluj-Napoca city, Romania (Figure 7). 

The concentrations of metals in poplar leaves increase along the vegetation period, being higher at senescence than at emergence [20,21]. Site 10 was considered a reference site as it is situated far from roads with high traffic in a green area. Sites 4, 6, 7, 9, and 11 are characterized by intense traffic, sites 1, 2, 3, 5, and 8 by moderate traffic, while sites 10 and 12 by low traffic. The sampled poplars were of similar height and similar age and did not show any illness or damage. In each site, 10–20 mature leaves from 3 branches from different sides of the outer canopy of a single tree were collected from a height of 2–2.5 m. Only leaves that were not affected by bird falls, insect infection, chlorosis, or necrosis were selected. The leaves were separated from the branches using a Teflon coated scissor and transported into the lab in paper bags.

In the same sites, instantaneous values of the TSP, particles with diameter less than 10 μm (PM_10_), 2.5 μm (PM_2.5_), and 1 μm (PM_1_) were measured in the ambient air using an Aerocet 831 handheld laser scattering particle counter (Met One Instruments, Washington, US). At each site, 25 consecutive measurements with the duration of 1 minute were performed and the results are reported as the average value. The Aerocet 831 counts and sizes particles in 7 different size ranges and then uses an algorithm to convert count data to mass measurements (µg/m^3^). It allows measurements in the 0–1000 µg/m^3^ concentration range with a resolution of 0.1 µg/m^3^ and accuracy of ± 10%. The precision and accuracy of direct reading particle-counters, such as Aerocet 831, were studied in detail by Borghi et al. [59]. The Aerocet 831 was successfully used in the evaluation of exposure levels to various airborne pollutants in Milan, Italy [60], to measure the concentrations of particulate matter around heavy traffic in Ogbomoso, Nigeria [61] and to assess the impact of various traffic congestion on Cupressus arizonica and *Pinus nigra* seed germination [62]. 

In sampling site 11, during a 7 day period, the PM_10_ was collected for 24 h on 150 mm diameter micro-glass fiber filters of MG227/1/60 grade (Sartorius, Gottingen, Germany) preconditioned (20 ± 2 °C and 45 ± 5% relative humidity) at a constant weight using a DHA-80 high volume sampler (Digitel Elektronik GmbH, Burs, Austria), with a flow rate of 500 L/min, equipped with an automatic sample charger. Unexposed filters were used as field and lab-blanks to control contamination during transportation and handling.

### 3.2. Sample Preparation and Analysis

The content of PM_10_ was determined gravimetrically, using a Cubis MSA125P analytical balance (Sartorius, Goettingen, Germany) after the filters conditioning to constant weight in a climatic chamber with constant relative humidity (45 ± 5%) and temperature (20 ± 2 °C) for 72 h.

The poplar leaves were washed with distilled water, blotted to dryness, freeze-dried at ‒40 °C and ‒25 psi using a FreeZone 2.5 Liter Benchtop freeze dry system (Labconco, KS, USA) and powdered with a GM200 Grindomix Knife Mill (Retsch GMBH, Haan, Germany).

Amounts of 1 g of leaf powder were digested with 5 mL of 65 % HNO_3_ and 2 mL of 30 % H_2_O_2_ in a closed polytetrafluoroethylene vessel MWS-3+ microwave digestion system (Berghof, Eningen, Germany). The digested samples were filtered and diluted to 25 mL with ultrapure water (Elga PURELAB flex, Veolia, Wycombe, UK). The filters loaded with PM_10_ were digested in an open vessel with 28 mL aqua regia (21 mL of 37 % HCl and 7 mL of 65% HNO_3_), at room temperature for 4 h and at reflux conditions for 2 h. After cooling to room temperature, the slurry was filtered and diluted to 25 mL with ultrapure water. The contents of Cu, Pb, Zn, Cd, Co, Ni, Fe, Mn, and Al in the digested samples were determined using an ELAN DRC II spectrometer (Perkin Elmer, Waltham, MA, United States). The calibration curves were prepared using 10 mg/L multielement standard solutions (Perkin Elmer, Waltham, MA, USA) ranging between 0–100 μg/L. Calibration standards, procedural blank measurements, and duplicate samples were used for the quality assurance of results. The accuracy of metal determination was checked by analyzing the NIST-SRM 1515 Apple leaves reference material. The mean recoveries for the determination of metals in CRMs ranged between 94% and 105%. The use of ICP-MS for the measurement of metal content in plants was reported also for poplar leaves, cinnamon tree leaves, and pine needles [16,33,63,64]. The metal concentrations were measured in duplicate and the results were reported as average.

### 3.3. Data Analysis

In order to identify the contribution of anthropogenic sources to the level of metals found in PM_10_, the enrichment factor (EF_PM10_) was calculated for each metal according to Equation (1), considering Al as a reference element, due to its minor contribution to the atmospheric pollution level [41]. Values of EF_PM10_ < 10 indicated that the metals were not enriched and had a crustal origin, 10 < EF_PM10_ > 100 indicated a moderate enrichment and a mixed origin of the elements, while EF_PM10_ > 100 indicated a high enrichment through anthropogenic sources [20,43,44,45].

(1) $$EF_{{PM}_{10}}=\frac{{\left(\frac{C_{M}}{C_{Al}}\right)}_{{PM}_{10}}}{{\left(\frac{C_{M}}{C_{Al}}\right)}_{UCC}}$$

where: (C_M_/C_Al_)_PM10_ is the ratio between the metal and Al concentration in PM_10_; (C_M_/C_Al_)_UCC_ is the ratio between the metal and Al concentration in the upper continental crust given by Rudnick and Gao [65].

To assess the ability of the poplar to simultaneously accumulate several metals, the metal accumulation index (MAI) was calculated according to Equation (2) [55].

(2) $$MAI=\frac{1}{N}\sum_{j=1}^{N}I_{j},$$

where: N is the number of metals (9 in our study); Ij is the ratio between the mean concentration of each metal and its standard deviation.

The enrichment factor of metals in poplar leaves (EF_poplar_) was calculated as the ratio of metal contents normalized to Al in the poplar leaves from the study and reference site according to Equation (3). EF_poplar_ < 1 indicated no enrichment, EF_poplar_ < 3 indicated minor enrichment, EF_poplar_ < 10 showed moderate enrichment, and EF_poplar_ > 10 suggested severe enrichment [40].

(3) $$EF_{poplar}=\frac{{\left(\frac{C_{M}}{C_{Al}}\right)}_{study\;site}}{{\left(\frac{C_{M}}{C_{Al}}\right)}_{reference\;site}},$$

where: (C_M_/C_Al_) is the ratio between the metal and Al concentration in poplar leaves.

The possible metal sources in the air were investigated using PCA. This method allows the reduction of the dataset into several independent components named PC’s. The varimax rotation approach on PC’s with eigenvalues higher than 1 was applied on standardized data using the XLSTAT (Addinsoft) Microsoft Excel add-on software. PCA analysis was previously used for metal sources identification in *Pinus eldarica* leaves and for the assessment of pollution trends in Adana urban area, Tukey, using the concentration of trace elements in the leaf samples collected from 75 trees [51,66].

## 4. Conclusions

Black poplar (*Populus nigra* L.) was used for the passive biomonitoring of air pollution with particulates in Cluj-Napoca city, Romania. High variability of the particulates and instantaneous PM_10_ density was found between the sampling sites, with values ranging between 38–134 μg/m^3^. PM_10_ was the major fraction of particulates (63%) in every site. The daily PM_10_ values ranged between 13–90 μg/m^3^ and contained only low levels of metals (<0.4%). The most abundant metals in PM_10_ were Al and Fe, followed by Zn, Mn, Cu, and Pb. The moderate enrichment factor of Cu, Pb, and Zn and the high enrichment factor of Cd in PM_10_ indicated that Cd is of anthropogenic origin, while the other metals have both natural and anthropogenic origin. In the poplar leaves samples, Zn was the major metal, followed by Fe, Mn, Al, Pb, Cu, Ni, Co, and Cd. The sum of metal contents in leaves increased with the increase of PM_10_. Metal accumulation in poplar leaves in measurable concentrations was observed in each site, for all studied metals, except for Cd, suggesting that poplar leaves are suitable to be used for the biomonitoring of pollution. The main identified anthropogenic sources of metals in the poplar leaves were: traffic and waste burning (Cu, Zn), road dust resuspension (Fe, Pb, Mn, Co), and soil contamination (Pb, Cu, Ni).

## Figures and Tables

**Table 1:** Concentration of heavy metals in the leaves of different species.

*Species*	*Heavy Metals (mg/kg)*									*Reference*
	*Cd*	*Co*	*Cu*	*Mn*	*Ni*	*Pb*	*Zn*	*Fe*	*Al*	
*Populus nigra* L.	1.60	1.10	4.60	36.9	2.00	3.50	119	59.4	32.7	Present study
*Pinus eldarica*	0.74	-	5.59	-	-	2.99	26.1	345	-	Miri et al. 2017 [51]
*Platanus orientalis* L.	-	-	15.1	-	-	1.13	35.0	404	-	Norouzi et al. 2015 [52]
*Ulmus umbraculifera*	0.08	-	7.97	-	2.76	1.55	22.5	-	-	Hajizadeh et al. 2019 [53]
*Juglans regia*	0.03	-	7.08	-	2.17	1.63	22.4	-	-	Hajizadeh et al. 2019 [53]
*Vitis vinifera*	0.03	-	5.11	-	2.10	1.24	24.8	-	-	Hajizadeh et al. 2019 [53]
*Taraxacum officinale*	1.29	-	10.1	24.8	-	28.1	180	159	-	Nadgorska-Socha et al. 2017 [54]
*Plantago lanceolata*	1.55	-	5.85	47.1	-	25.4	97.6	299	-	Nadgorska-Socha et al. 2017 [54]
*Betula pendula*	0.72	-	2.94	63.3	-	16.0	389	260	-	Nadgorska-Socha et al. 2017 [54]
*Robinia pseudoacacia*	0.65	-	1.78	25.2	-	11.1	52.7	192	-	Nadgorska-Socha et al. 2017 [54]

**Table 2:** Factor loadings of *PC*’s after varimax rotation.

	*PC1*	*PC2*	*PC3*	*PC4*
Eigenvalue	4.9	3.4	1.6	1.3
Variability (%)	38.3	25.9	12.3	10.2
Cumulative (%)	38.3	64.2	76.5	86.7
Fe	−0.002	**0.620 ^1^**	−0.070	**0.723**
Ni	0.082	−0.006	**0.918**	0.005
Co	−0.132	**0.736**	0.071	0.205
Cu	**0.762**	0.006	**0.568**	0.079
Zn	**0.915**	0.261	0.213	−0.090
Cd	−0.072	0.027	−0.416	**−** **0.776**
Pb	−0.105	**0.649**	**0.515**	0.081
Mn	0.265	**0.858**	−0.152	−0.030
Al	−0.122	0.154	−0.153	**0.913**
TSP	**0.700**	**0.602**	0.048	0.364
PM_10_	**0.800**	0.455	0.042	0.357
PM_2.5_	**0.943**	−0.182	−0.115	−0.133
PM_1_	**0.913**	−0.202	−0.052	−0.147

^1^ Bold data represent the elements with correlation values higher than 0.5.
